# Evaluation of Electrical and Optical Plethysmography Sensors for Noninvasive Monitoring of Hemoglobin Concentration

**DOI:** 10.3390/s120201816

**Published:** 2012-02-09

**Authors:** Justin P. Phillips, Michelle Hickey, Panayiotis A. Kyriacou

**Affiliations:** School of Engineering and Mathematical Sciences, City University London, Northampton Square, London EC1V 0HB, UK; E-Mails: Michelle.Hickey@city.ac.uk (M.H.); p.kyriacou@city.ac.uk (P.A.K.)

**Keywords:** hemoglobin, capacitance, plethysmography, noninvasive

## Abstract

Completely noninvasive monitoring of hemoglobin concentration has not yet been fully realized in the clinical setting. This study investigates the viability of measuring hemoglobin concentration noninvasively by evaluating the performance of two types of sensor using a tissue phantom perfused with a blood substitute. An electrical sensor designed to measure blood volume changes during the cardiac cycle was used together with an infrared optical sensor for detection of erythrocyte-bound hemoglobin. Both sensors demonstrated sensitivity to changes in pulse volume (plethysmography). The electrical sensor produced a signal referred to as capacitance plethysmograph (CPG) a quantity which was invariant to the concentration of an infrared absorbing dye present in the blood substitute. The optical sensor signal (photoplethysmograph) increased in amplitude with increasing absorber concentration. The ratio PPG:CPG is invariant to pulse pressure. This quantity is discussed as a possible index of *in vivo* hemoglobin concentration.

## 1. Introduction

Anemia is a common condition affecting approximately 2 billion people worldwide [1]. It is defined as a less than normal quantity of hemoglobin in the blood or a reduction in size and/or the number of red blood cells. The main function of hemoglobin is to carry oxygen from the lungs to the tissues, so anemia leads to hypoxia (lack of oxygen) in organs. Mild anemia is usually symptomless but in moderate cases, sufferers exhibit symptoms of tiredness and lethargy. Severe cases can lead to dizziness, shortness of breath or cardiac arrest. The clinical definition of anemia in adults is a blood concentration of hemoglobin of less than 12–13 mg/L of blood, usually manifested as a lack of red blood cells [2].

Currently, hemoglobin concentration measurement is performed by collecting a venous blood sample or a pinprick of blood from an accessible site such as a finger. The blood is transferred to a disposable cuvette which is then placed in a suitable analyzer and the hemoglobin concentration measured spectrophotometrically. Various factors are known to affect accuracy, many of which depend on individual sampling techniques [3,4].

The envisaged system, by contrast to this method would be completely pain-free and may be performed quickly and safely by an operator with minimal training (or by the patient in the home or other non clinical environment). There is no risk associated with handling blood and needles, and no need for disposal of biohazard waste. Furthermore, once the probe is applied to the patient, the readout may be continuous which would have useful application during surgery where significant blood loss can occur over a short timescale.

Estimation of hemoglobin concentration depends on the ability to detect the hemoglobin (or red cell) amount as well as the total blood volume (approximately red cells + plasma). Noninvasive estimation of hemoglobin concentration is possible, using recently-developed commercially available systems based on purely optical methods [5]. These systems show indeterminate accuracy and precision however [6], since the plasma component of the blood is optically transparent and is thus difficult to detect using optical methods alone.

Blood volume estimation in limbs is historically well-established using the technique of electrical impedance plethysmography [7]. Variations on the technique allow estimation of changes in volume in response to venous occlusion, pulse volume [8] and blood flow to be measured [9], although the technique has not found widespread clinical acceptance. It has been shown that the pulse signal as measured by electrical resistivity is sensitive to both blood volume changes (desirable) as well as changes in the resistivity of the blood itself (undesirable) although phase-dependent signal analysis is able to minimise these errors to the extent that the technique is still useable [10]. Capacitance-based sensors for measurement of blood flow *in vivo* have been described [11], as well as hematocrit (relative volume of red blood cells in blood) outside the body. Jaspard *et al.* described a method which showed that the permittivity of a fixed volume blood sample is strongly dependent on its hematocrit value [12], while others have described similar techniques for use in applications such as extra-corporeal circulation systems [13].

Prior to development of a single electro-optical sensor capable of measuring simultaneous time-dependent capacitance and absorbance variations, separate capacitance and optical sensors were developed. The capacitance sensor is sensitive to pulsatile variations in dielectric permittivity of the tissue. An optical photoplethysmography sensor was also developed to measure time-dependent absorbance variations. The two sensors were used to detect changes in capacitance and absorbance occurring in a tissue phantom consisting of elastic tubing simulating arteries filled with a saline and dye solution. Signals from both sensors are analyzed and compared for a range of dye concentrations and pulse pressures. The former investigation was designed to assess the system’s sensitivity to simulated changes in hemoglobin concentration while the latter was to assess any (undesirable) variation by factors other than hemoglobin concentration, namely blood pressure.

## 2. Materials and Methods

### 2.1. Capacitance Sensor and Instrumentation

**Figure 1.**
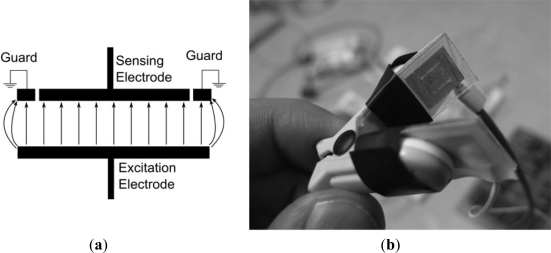
(**a**) Diagram of guarded capacitance sensor. (**b**) Photograph of sensor enclosed within earlobe probe shell.

The capacitance sensor [14] is essentially two plates of a parallel plate capacitor with so that when it is placed over an extremity, the tissue of the extremity forms the capacitor dielectric. A grounded guard rail was added as shown in Figure 1(a) which has the effect of reducing sideways ‘leakage’ of electric field at the edge of the sensor, *i.e.*, the field lines are effectively straightened at the plate edges. The plates were electrically insulated from the tissue using 0.1 mm thick adhesive cellulose film. The plates were attached to two limbs of a pulse oximeter probe shell as shown in the photograph in Figure 1(b). One plate (the excitation electrode) is attached to a signal generator supplying a 100 kHz sinusoidal signal of 1.5 V rms amplitude.

**Figure 2.**
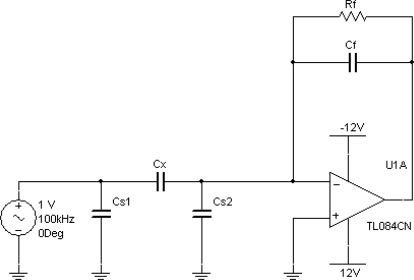
Circuit diagram of capacitance-voltage circuit for measuring the capacitance *Cx* of the probe.

The capacitance sensor was connected to a capacitance-to-voltage (C-V) circuit, shown in the diagram in Figure 2. It is based on a transimpedance amplifier, or current-to-voltage (I-V) converter, where the transimpedance is a capacitance. This circuit is used in electrical capacitance tomography systems. Any capacitive sensor has stray capacitances associated with it which are due to factors such as the screened cable connecting the sensing electrode to the measuring circuit (approximately 100 pF for a 1 metre cable). In the C-V circuit, the stray capacitance *C_s1_* is driven by the sine wave voltage source directly and will not affect capacitiance measurement. C*_s2_* is kept at virtual earth by the op-amp, therefore, there is no potential difference across it and no affect on capacitance measurement. Such a circuit is therefore considered, stray-capacitance immune.

*C_x_* is the capacitive sensor and *C_f_* is the feedback capacitance. *R_f_* is needed to bias the op-amp—to prevent the op-amp output drift which would eventually saturate the op-amp. The output of the circuit, *V_out_*, is given by:

(1) $$V_{\mathrm{out}}=\frac{j\omega C_{x}\;R_{f}}{j\omega C_{f}\;R_{f}+1}\;V_{\mathrm{in}}$$

where *ω* is the angular frequency of the sine-wave source. If |*jωC_f_R_f_*| >> 1 then

(2) $$V_{\mathrm{out}}=-\frac{C_{x}}{C_{f}}\;V_{\mathrm{in}}$$

The output voltage from the C-V circuit is amplified and the signal passed to a full-wave rectifier to demodulate the slowly-varying (dc) signal from the excitation signal. The rectified signal is low-pass filtered (cut-off frequency 22.5 Hz) to remove mains interference. The dc capacitance signal is tapped at this point, which is used to normalize the pulsatile (ac) capacitance signal. This signal is band pass filtered (0.1 Hz–22.5 Hz), and the resuting ac signal amplified (gain 54.5). The normalized (ac/dc) capacitance was calculated thus

(3) $$\frac{\Delta C_{x}}{C_{x}}=\frac{\Delta V_{\mathrm{out}}}{V_{\mathrm{out}}}$$

where Δ*V_out_*, is the ac voltage output from the capacitance measurement circuit and *V_out_*, is the dc voltage output. This quantity is referred to in the results section as the capacitance plethysmography (CPG) amplitude.

### 2.2. Optical Sensor and Instrumentation

The optical sensor was fabricated using a commercial earlobe pulse oximeter (GE Healthcare, Helsinki, Finland). The red-emitting light-emitting diode and PIN photodiode were utilized to make a single wavelength photoplethysmograph sensor. The LED was driven by a 24 mA dc signal, while the photodiode was connected to a transimpedance (I-V) amplifier of gain 6.67 × 10^5^ VA^−1^ and the signal lowpass filtered to produce a dc output. The signal was also bandpass filtered (0.4 Hz–15.9 Hz), and amplified to produce an ac output signal. The normalized (ac/dc) absorbance was calculated thus

(4) $$\frac{\Delta A}{A}=\frac{\Delta {V^{\prime }}_{\mathrm{out}}}{{V^{\prime }}_{\mathrm{out}}}$$

where Δ*V^′^_out_*, is the ac voltage output from the optical measurement circuit and *V^′^_out_*, is the dc voltage output. This quantity is referred to in the results section as the photoplethysmography (PPG) amplitude. The capacitance and optical signals were digitized and recorded using a National Instruments Data Acquisition card and recorded to a text file for later analysis using a LabVIEW virtual instrument.

### 2.3. *In Vitro* Model of Perfused Tissue

**Figure 3.**
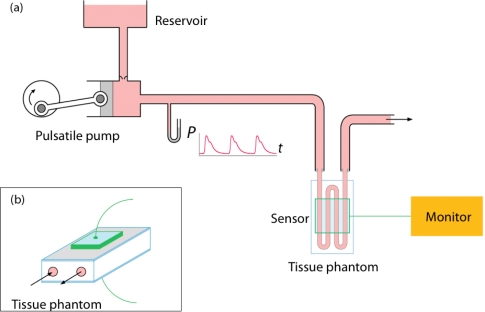
(**a**) Schematic diagram of *in vitro* circulation model and (**b**) tissue phantom.

A simple model of the circulation was constructed for the purposes of evaluating the performance of the electrical and optical sensors, which is shown schematically in Figure 3. A mixture of 0.9% saline containing black pigment dye was pumped using a custom pulsatile pump capable of generating a physiologic arterial pressure waveform of varying pulse pressure (systolic pressure minus diastolic pressure: selectable from 20 to 220 mmHg), at a constant frequency of 1 Hz (60 bpm ‘heart rate’). The pump was connected using a short length of stiff arterial pressure catheter tubing to a custom tissue phantom. The phantom consisted of a loop elastic silicone tubing of internal diameter 2.2 m enclosed within a glass cuvette of dimensions (*h* × *d* × *w)* 35 × 10 × 10 mm. The phantom is thus incompressible, being enclosed in glass. Besides the silicone tubing, the cuvette was otherwise empty, containing only air.

The flow rate through the system could be adjusted using a roller clamp downstream of the phantom. The electrical and optical sensors were attached to the phantom adjacent to one another as shown in Figure 3. This configuration allowed recording of simultaneous optical and capacitance measurements to be recorded.

### 2.4. Measurements: Effect of Variable Dye Concentration

The reservoir was filled with a saline solution containing dye with an optical absorbance (at 660 nm) equivalent to blood with a hemoglobin concentration of 60 g/dL, A Novaspec 4049 spectrophotometer (LKB-Produkter, Bromma, Sweden) was used to check the absorbance value. The pump was switched on and the tubing purged with the saline dye mixture. The flow rate was maintained at approximately 40 mL per minute through the system and the pulse pressure was maintained at 250 mmHg. Signals were recorded from the capacitance and optical probes for a period of 30 seconds. The normalized ‘capacitance plethysmograph’ (CPG) and photoplethysmograph (PPG) amplitudes were calculated from the acquired signals using Equations (3) and (4) and averaged over the 30 second measurement period. The dye concentration was adjusted by diluting the contents of the reservoir with an appropriate volume of saline and measurements repeated for a range of dye concentrations.

### 2.5. Measurements: Effect of Variable Pulse Pressure

A range of measurements were made using a similar protocol but with a constant dye concentration giving an optical absorbance equivalent to blood of hemoglobin concentration 20 g/dL. The pulse pressure however was varied from 20 mmHg to 220 mmHg, keeping diastolic pressure less than 20 mmHg (*i.e.*, the systolic pressure was increased). As before flow was maintained at approximately 40 mL/min.

## 3. Results

**Figure 4.**
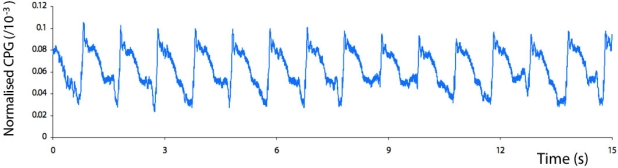
Sample of the time-varying capacitance plethysmograph (CPG) signal obtained when the systolic pressure was 250 mmHg and the dye concentration was equivalent to a hemoglobin concentration of 5 g/dL.

**Figure 5.**
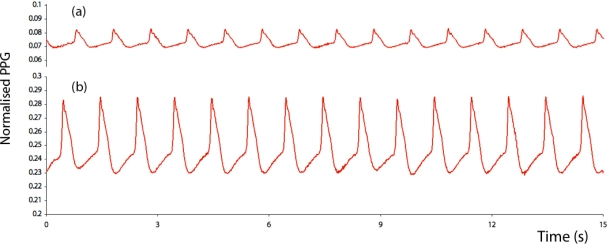
Samples of the time-varying photoplethysmograph (PPG) signal obtained when the systolic pressure was 250 mmHg and the dye concentration was equivalent to a hemoglobin concentration of (**a**) 5 g/dL, and (**b**) 20 g/dL.

It was found that the static capacitance of the probe and phantom was approximately 180 pF when the tubing within the phantom was filled with 0.9% saline. Figure 4 shows a sample of the time-varying capacitance plethysmograph (CPG) signal obtained when the systolic pressure was 250 mmHg and the dye concentration was equivalent to a hemoglobin concentration of 5 g/dL. Figure 5(a) shows a sample of the time-varying photoplethysmograph (PPG) signal obtained when the systolic pressure was 250 mmHg and the dye concentration was equivalent to a hemoglobin concentration of 5 g/dL, while Figure 5(b) shows the PPG signal at the same pressure but with a dye concentration equivalent to a hemoglobin concentration of 20 g/dL.

**Figure 6.**
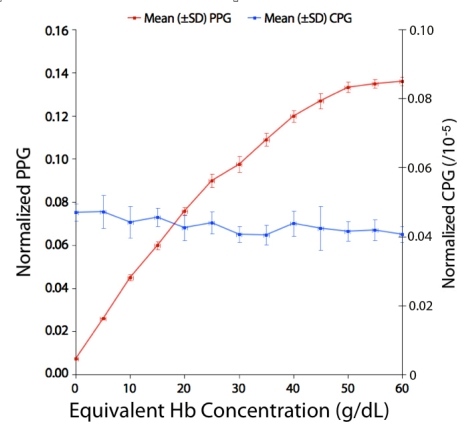
Graph of mean (±SD) normalized PPG amplitude (left *y*-axis) and mean (±SD) normalized CPG amplitude plotted against equivalent blood hemoglobin (Hb) concentration.

Figure 6 shows a graph of the peak-to-peak amplitudes of the normalized optical and capacitance signals (PPG and CPG respectively) plotted against equivalent hemoglobin concentration.

**Figure 7.**
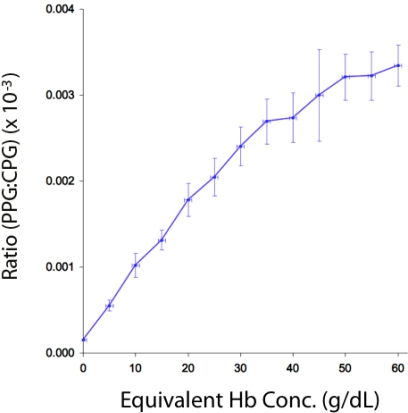
Graph of ratio of mean (±SD) normalized amplitudes (PPG:CPG ratio) plotted against equivalent blood hemoglobin (Hb) concentration.

It can be seen that the mean PPG amplitude increases greatly with increasing dye concentration. By contrast, the CPG amplitude changes little, decreasing very slightly, with increasing dye concentration. Figure 7 shows a graph of the ratio PPG:CPG amplitude (mean ± SD) against equivalent hemoglobin concentration. It can be seen that this ratio increases with increasing concentration.

**Figure 8.**
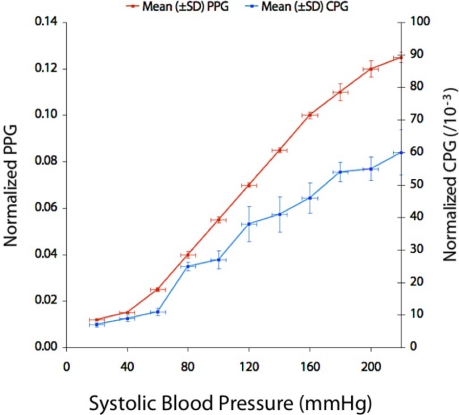
Graph of mean (±SD) normalized PPG amplitude (left *y*-axis) and mean (±SD) normalized CPG amplitude plotted against systolic pressure.

The following results were obtained from the second part of the study: an investigation of the effects of pulse pressure. Figure 8 shows a graph of the peak-to-peak amplitudes of the normalized optical and capacitance signals (PPG and CPG respectively) plotted against systolic pressure. Both quantities on the graph increase with increasing systolic blood pressure.

**Figure 9.**
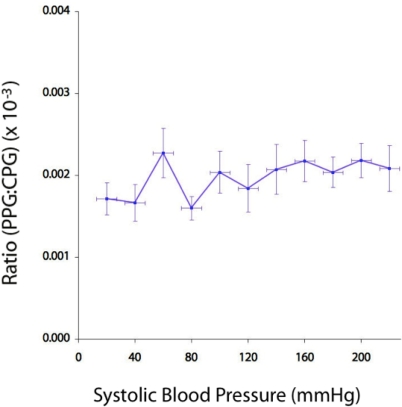
Graph of ratio of mean (±SD) normalized amplitudes (PPG:CPG ratio) plotted against pulse pressure.

Figure 9 shows a graph of the ratio PPG:CPG amplitude (mean ± SD) against pulse pressure. It can be seen that this ratio is not affected greatly by changes in pulse pressure. It can also be seen that the ratio varies considerably at low (<80 mmHg) pressures. This is thought to be due to low values of both PPG and CPG amplitude, resulting in relatively large variation in these quantities.

## 4. Discussion

This *in vitro* investigation demonstrated that simultaneous measurements of optical absorbance and capacitance can yield useful information regarding the composition of a blood substitute (saline and dye), namely the concentration of dye, which like hemoglobin and other chromophores is readily detectable using photoplethysmography. However, unlike hemoglobin, the plasma component of blood is more difficult to detect using optical methods alone, hence the need for a sensor based on a different measurement principle. The slight decrease in CPG amplitude with dye concentration could not be explained, but could be due to the addition of dye affecting the permittivity of the blood substitute. The ratio of PPG amplitude to CPG amplitude was seen to vary with varying dye concentration, which suggests that this value may be a useful index of hemoglobin concentration *in vivo*. The result is not surprising since the dye would not be expected to significantly affect the capacitance of the liquid component within the phantom. A major concern would be inter-patient variability caused by physiological factors other than hemoglobin concentration. For this reason the pulse pressure was chosen as an independent variable to further investigate this technique. It was found that the ratio PPG:CPG was invariant to pulse pressure, suggesting that if the technique were to be applied *in vivo*, the probe may be insensitive to blood pressure changes: a desirable property of a hemoglobin monitor.

Like pulse oximeters, a noninvasive hemoglobin monitor based on the technology described in this paper would be dependent on a measurable pulse signal, and therefore an adequate blood supply to the peripheral vessels. It could be supposed therefore that such a device may fail or produce unreliable readings under certain clinical conditions such as low blood pressure. The preliminary results suggest however, that as long as the finger or other tissue being monitored is well-perfused, the system will be sensitive to hemoglobin concentrations lower than the ‘safe’ clinical threshold (8–9 g/dL), although the lower limit of sensitivity will require experimental verification.

Further work is needed to validate the technique *in vivo* and to produce a noninvasive device for continuous monitoring of hemoglobin concentration. Early results suggest that the capacitance signal is small and difficult to resolve against background noise and interference in the signal. The instrumentation is currently undergoing revision and improved screening is being added to the measurement system in an attempt to mitigate this effect.
